# Police Killings and Police Deaths Are Public Health Data and Can Be Counted

**DOI:** 10.1371/journal.pmed.1001915

**Published:** 2015-12-08

**Authors:** Nancy Krieger, Jarvis T. Chen, Pamela D. Waterman, Mathew V. Kiang, Justin Feldman

**Affiliations:** Department of Social and Behavioral Sciences, Harvard T.H. Chan School of Public Health, Boston, Massachusetts, United States of America

## Abstract

Nancy Krieger and colleagues argue that law-enforcement–related deaths in the United States should be treated as notifiable conditions, which would allow public health departments to report these data in real-time.

**Summary Points**

- During the past year, the United States has experienced major controversies—and civil unrest—regarding the endemic problem of police violence and police deaths.
- Although deaths of police officers are well documented, no reliable official US data exist on the number of persons killed by the police, in part because of long-standing and well-documented resistance of police departments to making these data public.
- These deaths, however, are countable, as evidenced by “*The Counted*,” a website launched on June 1, 2015, by the newspaper *The Guardian*, published in the United Kingdom, which quickly revealed that by June 9, 2015, over 500 people in the US had been killed by the police since January 1, 2015, twice what would be expected based on estimates from the US Federal Bureau of Intelligence (FBI).
- Law-enforcement–related deaths, of both persons killed by law enforcement agents and also law enforcement agents killed in the line of duty, are a public health concern, not solely a criminal justice concern, since these events involve mortality and affect the well-being of the families and communities of the deceased; therefore, law-enforcement–related deaths are public health data, not solely criminal justice data.
- We propose that law-enforcement–related deaths be treated as a notifiable condition, which would allow public health departments to report these data in real-time, at the local as well as national level, thereby providing data needed to understand and prevent the problem.

## An Official Mystery: The United States Tally of Deaths Due to Police Violence (Yet Counted by a United Kingdom Newspaper)

During the past year, the United States has experienced major social controversies—and civil unrest—regarding police violence and police deaths [1,2]. Turning anger to action, the growing social movement *#Blacklivesmatter* has focused public attention on the long history and current realities of police brutality, both lethal and non-lethal, directed against the US black population [1,3].

Yet, although the number of US law enforcement agents killed in the line of duty is well documented (for 2015, 26 killed by shootings as of mid-September, of whom 17 were police officers)[2], no reliable official data exist on the number of US persons killed by the police [1]. On June 1, 2015, however, *The Guardian*-a newspaper from the United Kingdom—launched the “*The Counted*,” the first website that seeks to report, in real-time, the number of US people killed by police, and does so via “monitoring regional news outlets, research groups, and open-sourced reporting projects” as well as submissions from users [1] (see S1 Table for additional, albeit less comprehensive and less timely, sources). *The Counted*’s open data, extending back to January 1, 2015, include: (a) the decedent’s geographic location, gender, race/ethnicity, age, and photograph; (b) if the decedent was armed (if yes, with what kind of weapon); and (c) cause of death (“gunshot,” “taser,” “struck by vehicle,” “death in custody,” and “unknown”) [1]. Its data indicate that, as of October 6, 2015, 886 people in the US have been killed by the police since the year’s start (217 black, of whom 64, or 30%, were unarmed)[1]. Moreover, one week after its launch, it reported, on June 9, 2015, that the cumulative number of persons killed by police in the US had surpassed 500, twice what would be expected based on estimates of the US Federal Bureau of Investigation [1]. The website reports it will continue documenting data through the end of 2015; it is unknown if it will continue after this date [1].

It is startling that we, in the US, must rely on a UK newspaper for systematic timely counts of the number of persons killed by the police. After all, we have a world-class public health system that reports, nationally, in real-time, on numerous notifiable diseases and also on deaths occurring in 122 cities with populations >100,000 [4]. As of September 19, 2015, the cumulative 2015 total of 842 US persons killed by the police [1] notably exceeded the corresponding totals reported for the 122 cities’ 442 deaths under age 25 (all causes) and also 585 deaths (all ages) due to pneumonia and influenza, and likewise exceeded the national totals for several diseases of considerable concern: measles (188 cases), malaria (786 cases), and mumps (436 cases), and was on par with the national number of cases of Hepatitis A (890 cases) [4]. Just as epidemic outbreaks can threaten the public’s health, so too can police violence and impunity imperil communities’ social and economic well-being, especially if civil unrest ensues [1,3,5–8]. For example, in Baltimore, in late April 2015, in the wake of the death of Freddie Gray, a 25-year old African American man who was fatally injured while in the custody of the police, the resulting civil unrest, which occurred prior to charges being brought against the six police officers involved, led to immediate and long-term public health harms, including medication crises linked to the destruction of a dozen pharmacies, opioids from these pharmacies entering the illicit drug street market, mental health trauma, and further damage to the economies of neighborhoods already burdened by high rates of unemployment and premature mortality [5].

## A Public Health Solution: Make Law-Enforcement–Related Deaths a Notifiable Condition

Because reliable real-time data on law-enforcement–related deaths are critical for the public’s well-being, and because efforts over the past century to obtain data from law enforcement agencies on the number of deaths caused by police have been unsuccessful [3,8], we propose an alternative and already available policy route: make all law-enforcement–related deaths reportable conditions.

A core premise of our proposal is that mortality and morbidity due to police violence is a matter of public health, not just criminal justice [5–7,9,10], as is the occupational health of law-enforcement officials [2]. At issue are not only the direct harms to individuals but the toll taken on family members and communities, both for persons killed by the police and for police killed in the line of duty [1–3,5–10]. The role of public health is to document the deaths that have occurred; it is a separate matter, in the realm of the legal system, to determine the circumstances under which the deaths have occurred (e.g., whether use of force was justifiable or not) [3,8]. However, in addition to the harms experienced directly by individuals due to law-enforcement–related violence, there is another important casualty: the public health harms that arise from the damage rendered to the body politic itself [1,3,8]. Police are one of the most visible “faces” of government, whose work daily puts them in view of the public they are sworn to protect [3,8]. Combine excess police violence with inadequate prosecution of such violence, and the ties that bind citizens and their democratically elected governments become deeply frayed, with vicious cycles of distrust and violence fueling dysfunctional policing and dysfunctional governance more generally [1–3,5–8]. The direct effects and spill-over effects matter for public health and medicine alike, as reflected in the impact on emergency medical services, trauma units, mental health, and the trust required to deliver and implement any government-sponsored program, public health or otherwise [1–3,5–8].

**Fig 1:**
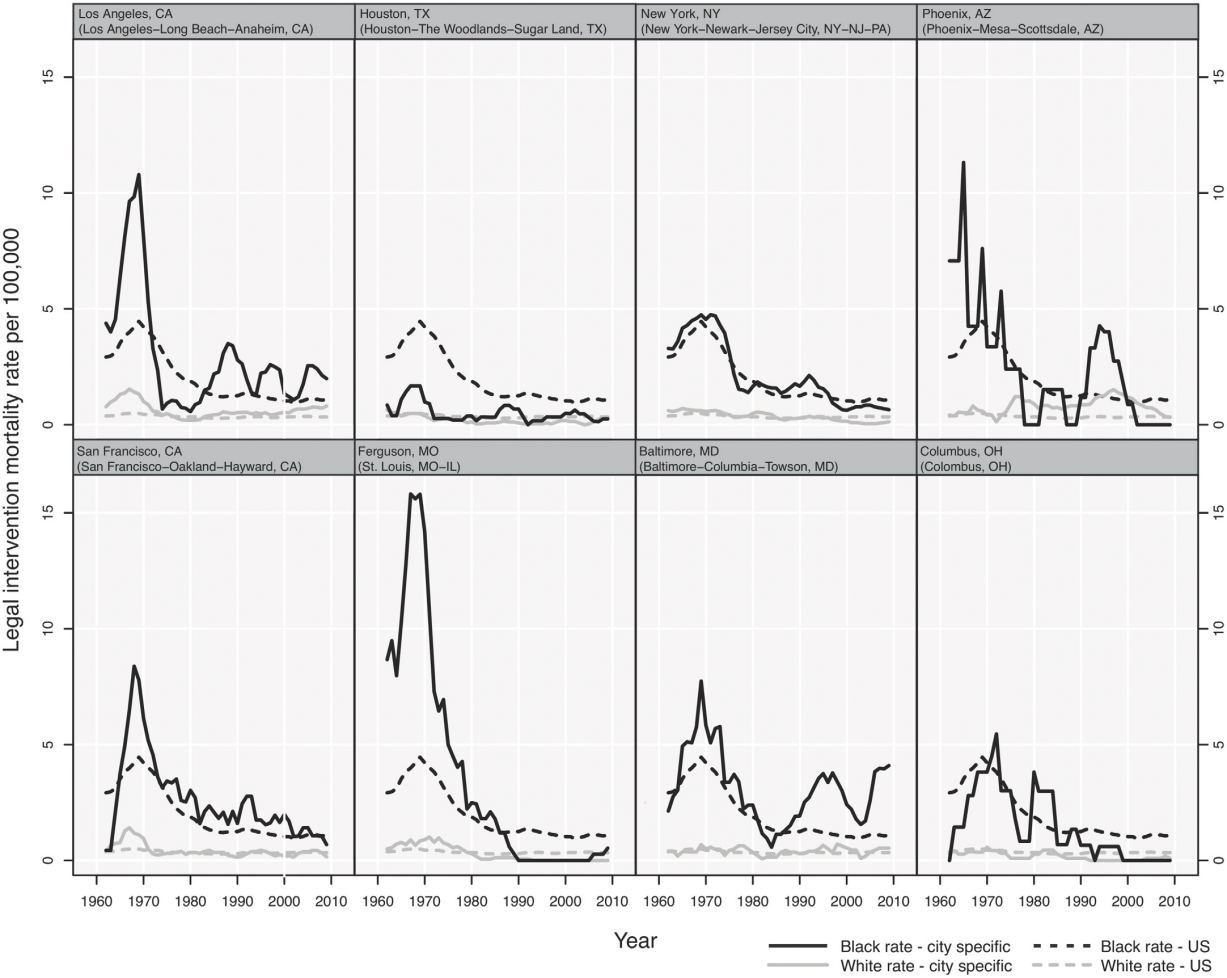
US deaths due to legal intervention: national and city-specific annual 5-year moving average rate (per 100,000) among US black men and white men ages 15–34, 1960–2011.

Further attesting to public health possibility of—and need for—timely and routine data on police killings are the data in Fig 1, which builds on our prior analyses of long-term trends in US deaths due to legal intervention (1960–2010) among black and white men, ages 15 to 34 [9]. The graphs show the rates (1960–2011, using 5-year moving averages) for eight cities, which we selected by searching *The Guardian* website [1] and picking the top five cities (as of June 12, 2015, shortly after the website was launched) for number of persons killed in 2015 by the police (Los Angeles, CA; Houston, TX; New York, NY; Phoenix, AZ; San Francisco, CA) plus the top three cities most mentioned in 2015 (in addition to these five cities) for protests against police violence (Ferguson, MO; Baltimore, MD; Cleveland, OH).

Calling for more detailed analysis, two patterns stand out:

1. The long-standing black versus white excess risk, which, despite declining over time, nationally remained 3.1 times higher (95% confidence interval [CI] 2.7, 3.5) in 2005, having been 7.8 times higher (95% CI 6.9, 8.8) in 1965 (S2 Table).
2. The variation within and across cities over time. For example, the black versus white rate ratio in 1965, 1985, and 2005 in New York City equaled, respectively, 6.0 (95% CI 4.2, 8.5), 5.3 (95% CI 3.4, 8.4), and 18.6 (95% CI 6.4,54.7); for Cleveland, these rate ratios were 18.9 (95% CI 7.5, 47.3), 13.6 (5.4, 34.6), and 6.3 (95% CI 2.1, 18.8) (S2 Table).

These patterns point to both the persistence of the problem—and the possibilities for change.

## Counting for Accountability: The Power of Public Health Data to Make Lives Matter

Three problems limit reliance on the publicly available US national mortality data: (1) they likely provide a conservative estimate (due to underreporting of police killings) [1,3,10]; (2) they are not available on a real-time basis [9,10]; and (3) they are aggregated to the county level [9,10]. Nor are these gaps filled by the US National Violent Death Reporting System (NVDRS), which presently includes only 32 states, and whose public access data are available only up through 2012 and only at the state level [11].

Hence our public health proposal to treat all law-enforcement–related deaths as a reportable health condition. No act of Congress is needed. No police department need be involved. Public health agencies can do the job. Public health experts, working with the US Council of State and Territorial Epidemiologists (which issues recommendations for notifiable conditions [4]) and with public input, can together create uniform case definitions and surveillance protocols to compile, in one uniform system, both: (a) deaths caused by law-enforcement officials (whether in the public or private sector, e.g., both local police officers and private security guards) and (b) occupational fatalities of law-enforcement officials. In our state of Massachusetts, for example, reportable conditions are governed by state regulations [12], they are allowed to include: “injuries or causes of injuries” pertaining to “assaults or homicides,” “strikes by/against another object or person,” “traumatic brain injuries,” and “weapons” ([12], p. 23) (Box 1), and triggers for investigating reportable conditions can include not only health records but also media reports [12], such as *The Counted* [1]. Although not yet used in Massachusetts to report law-enforcement–related deaths, the enabling regulations exist.

**Box 1. Injuries Included among Notifiable Health Conditions in Massachusetts [12]**

**p. 21: (E) Reporting of Work-Related Traumatic Injuries to a Person Less than 18 Years of Age**

1. By Health Care Facilities: Work-related traumatic injuries to persons less than 18 years of age that are treated in a hospital or other health care facility shall be reported by the person in charge of the facility or their designee. Health care facilities shall report these cases through computer-generated reports on a regular basis no less than once every six months. Said reports shall include similar information to that required under 300.180(D).
2. By Physicians and Other Health Care Providers: Serious work-related traumatic injuries to persons less than 18 years of age shall be reported to the Department by the physician or other health care provider who treats the minor, within 10 days after the physician or health care provider initially treats the injury. Physicians and other health care providers may report all work-related traumatic injuries to persons under 18 years of age. Said reports shall include similar information to that required under 300.180(D).

**p. 23: 300.193: Surveillance of Injuries Dangerous to Public Health**

The Department is authorized to collect medical records and other identifiable information from health care providers and other persons subject to 105 CMR 300.000 *et seq*., and/or prepare data, as detailed in 105 CMR 300.190 and 105 CMR 300.191, related to the following types of injuries or causes of injuries:

Assaults or homicides
Drownings
Falls
Fires
Machinery
Poisoning, including but not limited to, drug overdose
Spinal cord injuries
Strikes by/against another object or person
Suffocation
Suicides, attempted suicides, or self-inflicted wounds
Any mode of transportation
Traumatic amputations
Traumatic brain injuries
Weapons
Work-related injuries

Hence our proposal that law-enforcement–related deaths be a notifiable condition, reported in real time by medical and public health professionals. The harms to individuals and to the public’s health merit this monitoring. To our knowledge, this proposed course of action has not previously been suggested. The resulting data could inform advocacy within and across US states to reduce law-enforcement–related mortality and also set precedent for the more complex and costly task of monitoring law-enforcement–related injuries.

Underscoring the need for this public health approach is the new statement, on October 5, 2015, by the recently appointed US Attorney General Loretta Lynch, that the Department of Justice (DOJ) will begin piloting, in 2016, an open-source system akin to that used by *The Counted* to count the number of “officer-related deaths,” which would then “move towards verifying facts about the incident by surveying local police departments, medical examiner’s offices, and investigative offices” [13]. Timely public health data on all law-enforcement–related deaths, per that provided by the system of reportable notifiable conditions, will be all the more important for providing a credible source of data and verification, should the proposed DOJ pilot be successful and also sustainable past the upcoming presidential elections in November 2016.

It is stunning that we in the US must turn to a UK newspaper website [1] for timely and detailed reporting on deaths due to police violence. It also is unnecessary. A policy mechanism already exists. It is time that public health agencies exercise their ability to report to the public, in a timely manner, vital data on law-enforcement–related mortality that are critical to the well-being of communities and the body politic itself.

## Data Presented in the Paper

The data from *The Guardian* website are publicly available at [1]; the data from the US notifiable disease reporting system are available at [4]; the data on US deaths due to legal intervention from the US Compressed Mortality File are public access data that investigators can obtain solely and directly from the US National Center for Health Statistics (NCHS) and which may not be publicly shared, as explained in [9].

## Supporting Information

S1 Table Sources reporting law-enforcement–related deaths in the US. (DOCX)

S2 Table US deaths due to legal intervention: national and city-specific 5-year average annual rate (per 100,000) among US black men and white men ages 15–34, and rate ratios and differences: 1965, 1975, 1985, 1995, and 2005. (DOCX)
