# Supplementary material for: Police Killings and Police Deaths Are Public Health Data and Can Be Counted
Source: PLoS Med. 2015 Dec 8;12(12):e1001915. doi: 10.1371/journal.pmed.1001915 (PMC4672939; doi:10.1371/journal.pmed.1001915)
Supplement: S1 Table — (DOCX) [file pmed.1001915.s001.docx]

**S1 Table. Sources reporting law-enforcement related deaths in the United States.**

| **Type of source** | **Name and website^a^** |
| --- | --- |
| **1) News agency** | *The Guardian:* *The Counted: people killed by police in the US*  -- website: [http://www.theguardian.com/us-news/ng-interactive/2015/jun/01/the-counted-police-killings-us-database#](http://www.theguardian.com/us-news/ng-interactive/2015/jun/01/the-counted-police-killings-us-database)  -- Features real-time and detailed data, inclusive of any cause of death due to police action (e.g., gun, Taser, physical force, etc), starting as of January 1, 2015 (N as of September 15, 2015: 823 deaths). A description of how and why the website collects data is available at: <http://www.theguardian.com/us-news/ng-interactive/2015/jun/01/about-the-counted>  Relevant information as to its sources is as follows:  -- “The database will combine Guardian reporting with verified crowdsourced information to build a more comprehensive record of such fatalities. The Counted is the most thorough public accounting for deadly use of force in the US, but it will operate as an imperfect work in progress – and will be updated by Guardian reporters and interactive journalists as frequently and as promptly as possible.”  -- “… we count with traditional reporting on police reports and witness statements, by monitoring regional news outlets, research groups and open-source reporting projects such as the websites [Fatal Encounters](http://www.fatalencounters.org/) and [Killed by Police](http://killedbypolice.net/). But our intention is to progress to a verified crowdsourced system … We welcome all contributions of information that may improve the quality of our data. The Counted is a comprehensive and ongoing process of verification, as we continue to work from an inaccurate standard toward a more perfect standard.”  The rationale provided for the creation of this website is the lack of reliable and official US data on the number of police killings. As stated by the website:  “The FBI runs a voluntary program through which law enforcement agencies may or may not choose to submit their annual count of “justifiable homicides”, which it defines as “the killing of a felon in the line of duty”. This system is arguably less valuable than having no system at all: fluctuations in the number of agencies choosing to report figures, plus faulty reporting by agencies that do report, have resulted in partially informed news coverage pointing misleadingly to trends that may or may not exist … The FBI system counted 461 justifiable homicides by law enforcement in 2013, the latest year for which data is available. Crowdsourced counts found almost 300 additional fatalities during that year. The Counted, upon its launch on June 1, 2015, had already found close to that number of killings in just the first five months of this year.”  The operation of this website may be only for 2015, noting that *The Guardian* states:  “At present, the Guardian is collecting data on those killed by police specifically in 2015.” (see: <http://www.theguardian.com/us-news/ng-interactive/2015/jun/01/about-the-counted>). |
|  | *The Washington Post* (episodic reports)  -- website: <http://www.washingtonpost.com/graphics/national/police-shootings/>  -- Features real-time data for “only shootings in which a police officer, while on duty, shot and killed a civilian,” starting as of January 1, 2015 (N as of October 6, 2015: 756 deaths). |
| **2) Advocacy group** | Fatal Encounters  -- website: <http://www.fatalencounters.org/>  -- According to the website, **“This database contains records of people who’ve been killed through interactions with law enforcement since Jan. 1, 2000. At about 40 percent of its eventual size, it’s far from comprehensive. We anticipate completion of the national database by August 2017.”** |
|  | The Cato Institute’s National Police Misconduct Reporting Project  -- website: <http://www.policemisconduct.net/statistics/>  -- issues quarterly reports (including data on fatalities) in 2009 and 2010 only, |
|  | The National Law Enforcement Memorial Fund.  -- website: <http://nleomf.com/>  -- “serves as a nationwide clearinghouse of information and statistics on law enforcement line-of-duty deaths” (N as of October 6, 2015: 98 fatalities, 30 firearm related, 42 traffic-related, 26 other causes) |
| **3) US vital statistics** | US Centers for Disease Control and Prevention (for deaths due to legal intervention)  a) Compressed mortality data (publicly available county-level data back, for 1968-2013)  -- website: <http://wonder.cdc.gov/mortSQL.html>  b) CDC Wonder detailed mortality data (publicly available county-level data, for 1999-2013)  -- website: <http://wonder.cdc.gov/ucd-icd10.html>  c) National Violent Death Reporting System (publicly available state level data for 32 states, presently covering 2003-2012)  -- website: <http://www.cdc.gov/ViolencePrevention/NVDRS/> |
| **4) US Department of Justice** | Bureau of Justice Statistics  -- website: <http://www.bjs.gov/index.cfm?ty=tp&tid=7>  -- According to the website: (a) “The BJS Law Enforcement Unit maintains more than a dozen national data collections covering federal, state, and local law enforcement agencies and special topics in law enforcement. Most data series are collected every 2 to 4 years and focus on aggregate or agency-level responses, meaning the information that is collected pertains to units, such as police departments, training academies, and crime labs. The data from law enforcement agencies provide national estimates for personnel, equipment, operations, agency policies, budgets, and job functions across agencies. The Law Enforcement Unit collects data on contacts between the public and police, special units within law enforcement agencies, and trends in police organization.”; and (b) the most recent data reported are for 2013.  -- On October 5, 2015, the recently appointed US Attorney General Loretta Lynch announced that the DOJ would start piloting, in 2016, an open-source method of tracking “officer-involved deaths,” akin to that employed by “The Counted,” with the intent of “verifying the facts about the incident by surveying local police departments, medical examiner’s offices and investigative offices” (http://www.theguardian.com/us-news/2015/oct/05/justice-department-trials-system-count-killings-us-law-enforcement-the-counted). |
| **5) US Federal Bureau of Investigation** | Unified Crime Reporting Program: Supplementary Homicide Reports (SHR)  -- website: <https://www.fbi.gov/about-us/cjis/ucr/crime-in-the-u.s/2012/crime-in-the-u.s.-2012/offenses-known-to-law-enforcement/expanded-homicide/expandhomicidemain>  -- According to website: “The FBI’s Uniform Crime Reporting (UCR) Program collects supplementary homicide data that provide the age, sex, and race of the murder victim and offender; the type of weapon used; the relationship of the victim to the offender; and the circumstance surrounding the incident. Statistics gleaned from these supplemental data are provided in this section.”  -- Collects data on “justifiable homicides” by law enforcement officers-- Dataset available at: <http://www.icpsr.umich.edu/icpsrweb/NACJD/studies/36124>  -- Limitations are that reporting of data for the SHR to the FBI is voluntary and, in the case of homicides, the US Department of Justice has documented that many cases are not reported or having missing information (see: <http://www.bjs.gov/index.cfm?ty=pbdetail&iid=5259>) |
| ^a^ all websites accessed on October 6, 2015. | |
