# Supplementary material for: Police Killings and Police Deaths Are Public Health Data and Can Be Counted
Source: PLoS Med. 2015 Dec 8;12(12):e1001915. doi: 10.1371/journal.pmed.1001915 (PMC4672939; doi:10.1371/journal.pmed.1001915)
Supplement: S2 Table — (DOCX) [file pmed.1001915.s002.docx]

| **S2 Table. US deaths due to legal intervention^a^: national and city-specific^b^ 5-year average annual rate (per 100,000) among US black men and white men ages 15-34, and rate ratios and differences: 1965, 1975, 1985, 1995, and 2005.^c^** | | | | | | | | | |
| --- | --- | --- | --- | --- | --- | --- | --- | --- | --- |
| Year | Location | Rate per 100,000  (95% Confidence interval [CI]) | | | | Black vs. White | | | |
|  |  | Black | | White | | Rate Ratio  (95% CI) | | Rate Difference (95% CI) | |
| 1965 | US | 3.53 | (3.22, 3.84) | 0.45 | (0.41, 0.49) | 7.81 | (6.91, 8.83) | 3.08 | (2.77, 3.39) |
|  | Los Angeles‐Long Beach‐Anaheim, CA | 6.45 | (4.22, 8.68) | 1.32 | (0.99, 1.64) | 4.89 | (3.20, 7.48) | 5.13 | (2.87, 7.39) |
|  | Houston‐The Woodlands‐Sugar Land, TX | 1.12 | (-0.15, 2.39) | 0.37 | (0.01, 0.73) | 3.04 | (0.68, 13.56) | 0.75 | (-0.57, 2.07) |
|  | New York‐Newark‐Jersey City, NY‐NJ‐PA | 4.18 | (3.13, 5.23) | 0.70 | (0.53, 0.87) | 5.97 | (4.20, 8.49) | 3.48 | (2.42, 4.54) |
|  | Phoenix‐Mesa‐Scottsdale, AZ | 11.58 | (-1.52, 24.68) | 0.53 | (-0.07, 1.13) | 21.82 | (4.40, 108.09) | 11.05 | (-2.07, 24.16) |
|  | San Francisco‐Oakland‐Hayward, CA | 3.81 | (1.32, 6.30) | 0.78 | (0.37, 1.19) | 4.87 | (2.11, 11.26) | 3.03 | (0.51, 5.55) |
|  | St. Louis, MO‐IL | 10.41 | (6.06, 14.76) | 0.77 | (0.29, 1.25) | 13.45 | (6.37, 28.40) | 9.64 | (5.26, 14.02) |
|  | Baltimore‐Columbia‐Towson, MD | 4.98 | (2.46, 7.50) | 0.38 | (0.01, 0.76) | 13.02 | (4.32, 39.24) | 4.60 | (2.05, 7.15) |
|  | Cleveland‐Elyria, OH | 9.27 | (5.10, 13.44) | 0.49 | (0.10, 0.88) | 18.88 | (7.54, 47.28) | 8.78 | (4.59, 12.97) |
| 1975 | US | 2.80 | (2.57, 3.03) | 0.39 | (0.36, 0.42) | 7.25 | (6.46, 8.13) | 2.41 | (2.18, 2.64) |
|  | Los Angeles‐Long Beach‐Anaheim, CA | 0.95 | (0.25, 1.66) | 0.47 | (0.31, 0.64) | 2.02 | (0.89, 4.59) | 0.48 | (-0.24, 1.21) |
|  | Houston‐The Woodlands‐Sugar Land, TX | 0.24 | (-0.23, 0.70) | 0.26 | (0.03, 0.49) | 0.92 | (0.11, 7.87) | ‐0.02 | (-0.54, 0.50) |
|  | New York‐Newark‐Jersey City, NY‐NJ‐PA | 3.05 | (2.29, 3.80) | 0.43 | (0.30, 0.55) | 7.11 | (4.86, 10.39) | 2.62 | (1.86, 3.38) |
|  | Phoenix‐Mesa‐Scottsdale, AZ | 2.42 | (-2.32, 7.15) | 0.93 | (0.35, 1.51) | 2.60 | (0.33, 20.30) | 1.49 | (-3.28, 6.26) |
|  | San Francisco‐Oakland‐Hayward, CA | 3.40 | (1.39, 5.41) | 0.31 | (0.08, 0.55) | 10.81 | (4.19, 27.89) | 3.09 | (1.06, 5.11) |
|  | St. Louis, MO‐IL | 4.98 | (2.37, 7.59) | 0.68 | (0.28, 1.08) | 7.35 | (3.34, 16.19) | 4.30 | (1.66, 6.94) |
|  | Baltimore‐Columbia‐Towson, MD | 3.33 | (1.65, 5.02) | 0.44 | (0.09, 0.79) | 7.60 | (2.95, 19.60) | 2.90 | (1.17, 4.62) |
|  | Cleveland‐Elyria, OH | 11.05 | (7.03, 15.07) | 0.96 | (0.46, 1.47) | 11.45 | (6.05, 21.67) | 10.08 | (6.03, 14.14) |
| 1985 | US | 1.27 | (1.13, 1.41) | 0.28 | (0.25, 0.30) | 4.57 | (3.97, 5.26) | 0.99 | (0.85, 1.13) |
|  | Los Angeles‐Long Beach‐Anaheim, CA | 2.19 | (1.26, 3.13) | 0.43 | (0.29, 0.57) | 5.13 | (2.99, 8.81) | 1.77 | (0.82, 2.72) |
|  | Houston‐The Woodlands‐Sugar Land, TX | 0.32 | (-0.13, 0.77) | 0.07 | (-0.03, 0.17) | 4.43 | (0.62, 31.46) | 0.25 | (-0.21, 0.71) |
|  | New York‐Newark‐Jersey City, NY‐NJ‐PA | 1.58 | (1.11, 2.05) | 0.30 | (0.19, 0.40) | 5.31 | (3.36, 8.39) | 1.28 | (0.80, 1.76) |
|  | Phoenix‐Mesa‐Scottsdale, AZ | 1.44 | (-1.39, 4.28) | 0.76 | (0.33, 1.19) | 1.90 | (0.25, 14.64) | 0.69 | (-2.18, 3.55) |
|  | San Francisco‐Oakland‐Hayward, CA | 2.08 | (0.64, 3.53) | 0.35 | (0.11, 0.59) | 5.98 | (2.24, 15.93) | 1.74 | (0.27, 3.20) |
|  | St. Louis, MO‐IL | 1.79 | (0.36, 3.22) | 0.12 | (-0.05, 0.28) | 15.01 | (3.03, 74.36) | 1.67 | (0.23, 3.11) |
|  | Baltimore‐Columbia‐Towson, MD | 1.13 | (0.23, 2.04) | 0.14 | (-0.06, 0.34) | 7.93 | (1.60, 39.30) | 0.99 | (0.06, 1.92) |
|  | Cleveland‐Elyria, OH | 5.92 | (3.11, 8.73) | 0.43 | (0.09, 0.78) | 13.65 | (5.38, 34.61) | 5.49 | (2.65, 8.32) |
| 1995 | US | 1.20 | (1.07, 1.34) | 0.35 | (0.32, 0.38) | 3.47 | (3.03, 3.98) | 0.86 | (0.72, 0.99) |
|  | Los Angeles‐Long Beach‐Anaheim, CA | 2.19 | (1.18, 3.20) | 0.47 | (0.32, 0.63) | 4.62 | (2.63, 8.11) | 1.72 | (0.69, 2.74) |
|  | Houston‐The Woodlands‐Sugar Land, TX | 0.17 | (-0.16, 0.50) | 0.04 | (-0.04, 0.11) | 4.47 | (0.28, 71.53) | 0.13 | (-0.21, 0.47) |
|  | New York‐Newark‐Jersey City, NY‐NJ‐PA | 1.56 | (1.10, 2.02) | 0.29 | (0.18, 0.40) | 5.44 | (3.37, 8.79) | 1.27 | (0.80, 1.75) |
|  | Phoenix‐Mesa‐Scottsdale, AZ | 4.05 | (0.08, 8.03) | 1.26 | (0.77, 1.76) | 3.21 | (1.12, 9.23) | 2.79 | (-1.21, 6.80) |
|  | San Francisco‐Oakland‐Hayward, CA | 1.80 | (0.36, 3.25) | 0.44 | (0.15, 0.73) | 4.10 | (1.46, 11.53) | 1.36 | (-0.11, 2.84) |
|  | St. Louis, MO‐IL | 0.00 | (0.00, 0.00) | 0.00 | (0.00, 0.00) | --- | --- | 0.00 | (0.00, 0.00) |
|  | Baltimore‐Columbia‐Towson, MD | 3.75 | (2.06, 5.44) | 0.66 | (0.20, 1.11) | 5.70 | (2.49, 13.02) | 3.09 | (1.35, 4.84) |
|  | Cleveland‐Elyria, OH | 2.59 | (0.67, 4.51) | 0.70 | (0.22, 1.19) | 3.68 | (1.33, 10.15) | 1.89 | (-0.09, 3.87) |
| 2005 | US | 1.11 | (0.99, 1.23) | 0.36 | (0.33, 0.39) | 3.07 | (2.68, 3.51) | 0.75 | (0.63, 0.87) |
|  | Los Angeles‐Long Beach‐Anaheim, CA | 2.55 | (1.40, 3.69) | 0.70 | (0.51, 0.89) | 3.62 | (2.14, 6.12) | 1.84 | (0.68, 3.00) |
|  | Houston‐The Woodlands‐Sugar Land, TX | 0.29 | (-0.11, 0.69) | 0.03 | (-0.03, 0.10) | 8.69 | (0.79, 95.86) | 0.26 | (-0.15, 0.66) |
|  | New York‐Newark‐Jersey City, NY‐NJ‐PA | 0.85 | (0.50, 1.19) | 0.05 | (0.00, 0.09) | 18.58 | (6.43, 53.73) | 0.80 | (0.45, 1.15) |
|  | Phoenix‐Mesa‐Scottsdale, AZ | 0.00 | (0.00, 0.00) | 0.73 | (0.39, 1.06) | 0.00 | --- | ‐0.73 | (-1.06, ‐0.39) |
|  | San Francisco‐Oakland‐Hayward, CA | 1.42 | (0.03, 2.80) | 0.37 | (0.10, 0.65) | 3.78 | (1.11, 12.93) | 1.04 | (-0.37, 2.46) |
|  | St. Louis, MO‐IL | 0.00 | (0.00, 0.00) | 0.00 | (0.00, 0.00) | --- | --- | 0.00 | (0.00, 0.00) |
|  | Baltimore‐Columbia‐Towson, MD | 2.63 | (1.25, 4.01) | 0.36 | (0.01, 0.71) | 7.33 | (2.41, 22.26) | 2.27 | (0.85, 3.70) |
|  | Cleveland‐Elyria, OH | 3.26 | (1.13, 5.39) | 0.52 | (0.06, 0.97) | 6.31 | (2.11, 18.82) | 2.74 | (0.57, 4.92) |
| ^a^ Classifications employed for “death due to legal intervention”:   \| **International Classification of Diseases (ICD): version*** \| **Death due to legal intervention** \| \| --- \| --- \| \| ICD-7 (1958-1967) \| (E984) “Injury by intervention of police” (and not including: (E985) “Execution”) \| \| ICD-8 (1968-1978) \| (E970-E977) “Legal intervention” (and excluding (E978)”Legal execution”) \| \| ICD-9 (1979-1998) \| (E970-E977) “Legal intervention (and excluding (E978) “Legal execution”) \| \| ICD-10 (1999-20120) \| (Y35.0-Y35.4, Y35.6-Y35.7) “legal intervention” (and excluding (Y35.5) “executions”) \| \| * ICD codes as listed at: <http://www.wolfbane.com/icd/> \| \|   ^b^ Cities selected using *The Guardian* website (*1*) and employ data for the US-census defined “core based statistical area” (CBSA; see: <http://www.census.gov/population/metro/>) in which each city is located, and include (a) the 5 US cities with the highest number of persons killed (as of June 12, 2015) by the police (Los Angeles, CA; Houston, TX; New York, NY; Phoenix, AZ; San Francisco, CA), and (b) the 3 cities most named (in addition to these 5 cities) as having large-scale police protests in 2015 (Ferguson, MO; Baltimore, MD; Cleveland, OH).  ^c^ Source of data: US Compressed Mortality File (see, for mortality during and after 1968, <http://www.cdc.gov/nchs/data_access/cmf.htm>; see also reference 9 regarding the 1960-1967 US mortality file). | | | | | | | | | |
